# Early detection of novel *Leishmania* species DNA in the saliva of two HIV-infected patients

**DOI:** 10.1186/s12879-016-1433-2

**Published:** 2016-02-24

**Authors:** Padet Siriyasatien, Sarunyou Chusri, Kanyarat Kraivichian, Narissara Jariyapan, Thanaporn Hortiwakul, Khachornsakdi Silpapojakul, Adam M. Pym, Atchara Phumee

**Affiliations:** Department of Parasitology, Faculty of Medicine, Chulalongkorn University, Bangkok, 10330 Thailand; Excellence Center for Emerging Infectious Disease, King Chulalongkorn Memorial Hospital, Thai Red Cross Society, Bangkok, 10330 Thailand; Division of Infectious Diseases, Department of Internal Medicine, Faculty of Medicine, Prince of Songkla University, Songkhla, 90110 Thailand; Department of Parasitology, Faculty of Medicine, Chiang Mai University, Chiang Mai, 50200 Thailand; School of Life Sciences, University of Liverpool, Liverpool, UK

**Keywords:** *Leishmania*, Saliva, HIV-infection, PCR

## Abstract

**Background:**

Leishmaniasis caused by two new species of *Leishmania*; *L. siamensis* and *L. martiniquensis* have been recently described in Thailand. The disease has mainly been documented in AIDS patients from southern Thailand. In this study, polymerase chain reaction (PCR) was used to determine HIV-*Leishmania* co-infection in southern Thailand.

**Methods:**

One ml of saliva and 3 ml of EDTA blood were collected from HIV-infected patients for PCR detection of *Leishmania* DNA, cloning and sequencing. The positive PCR samples were then cultured on Schneider’s insect medium.

**Results:**

Three out of 316 saliva samples collected from HIV-infected patients were found to be positive for *Leishmania* DNA (0.95 %). Among the positive samples, one patient was observed with disseminated cutaneous lesions and also tested positive via saliva, whole blood and buffy coat in PCR. The second case presenting with nodular lesions also gave a positive saliva test via PCR two months prior to buffy coat. This diagnosis was confirmed by microscopic examination and a culture of biopsy samples from a nodule. The last case was an asymptomatic *Leishmania* infection which tested PCR positive only in saliva with a consecutive sample collection conducted for three months.

**Conclusions:**

The prevalence of *Leishmania* infection in HIV infected patients within this study is 0.95 %. *Leishmania* DNA was detected in saliva by PCR prior to blood and buffy coat of two HIV infected patients. Early detection of *Leishmania* DNA in saliva would be beneficial for the follow up of asymptomatic *Leishmania* infected patients, the early treatment of leishmaniasis and for surveillance survey purpose. However, full evaluation of sensitivity and specificity of this technique with a large cohort of patients is required before deployment.

## Background

Autochthonous leishmaniasis cases in Thailand have been increasingly diagnosed in recent years. The disease was described in both immunocompetent and immunocompromised patients, such as those with AIDS [1] and in systemic steroid therapy [2]. Approximately 20 cases of autochthonous leishmaniasis have now been documented, with most found in the south of Thailand [1–9]. Sukmee and others first reported a suspected new *Leishmania* species from Thailand (2008) [3] which was named *L. siamensis* [7]. A report by Leelayoova et al. (2013) [10] demonstrated that *L. siamensis* in Thailand has two lineages: the PG lineage or PCM1 isolate (Accession no JX195640) [3] and TR lineage or PCM2 isolate (Accession no EF200012) [7]. However, more recently Pothirat et al. (2014) [11] identified a PCM1 and a new isolate from northern Thailand LSCM1 (Accession no JX898938) which are *L. martiniquensis* as described by Desbois et al. (2014) [12], and only the PCM2 isolate was identified as *L. siamensis*. They also reiterated that most cases of leishmaniasis are caused by *L. martiniquensis* in Thailand. More recently, Chiewchanvit et al. (2015) also described a case of HIV and *L. martiniquensis* co-infection in northern Thailand who presented with chronic generalized fibrotic skin lesions [13].

In other parts of the world such as isolates from Myanmar patients (Accession no KF211417) [2], cows in Switzerland (Accession no GQ281282), a horse in Germany (Accession no GQ281278) and a horse in the USA (Accession no JQ617283) [14–16] may be *L. martiniquensis*. Liautaud et al. (2015) reported the first case of visceral leishmaniasis caused by *L. martiniquensis* from the Caribbean [17]. This indicates that *L. martiniquensis* has a worldwide distribution while *L. siamensis* is limited in its geographic distribution.

Three clinical forms of these novel *Leishmania* species have been described: visceral, disseminated cutaneous, and combined disseminated cutaneous with visceral [1–9, 11, 13]. The disease has been described mostly in immunocompromised patients, especially those with AIDS. Apart from *L. martiniquensis* and *L. siamensis*, an autochthonous leishmaniasis case caused by *L. infantum* was also reported from Thailand [18].

The prevalence of leishmaniasis in Thailand has never been fully studied. Screening tests for leishmaniasis, such as Enzyme-linked immunosorbent assay (ELISA), Direct antiglobulin test (DAT) and rK39 dipsticks, are not generally available. Microscopic examination and culture are time-consuming and require expertise to be reliable. Microscopy, culture and PCR are generally the methods of choice used for diagnosis [1].

PCR has been developed to detect *Leishmania* DNA, and *Leishmania* species were identified by a sequence analysis [19–21]. PCR has high sensitivity and specificity for detecting *Leishmania* DNA [22, 23] and has been used for detection from various clinical samples including blood, bone marrow, tissue, saliva, and urine [1–6]. Saliva has been shown to be a good source for the detection of the new *Leishmania* species DNA [1–6].

Several previous studies demonstrated that *Leishmania* DNA and antibodies were present in oral secretions and saliva, such as *L. braziliensis* DNA from Brazil [24], *L. donovani* from China [25] and *L. infantum* from Tunisia [26]. In Thailand, Phumee et al. (2013) demonstrated that saliva is a good source for PCR detection of novel *Leishmania* species DNA in Thailand [1, 2, 4–6, 9]. They also showed that the *Leishmania* DNA levels in saliva decreased after treatment [1]. Saliva could be used as a biomarker to detect the new *Leishmania* species infection. Furthermore, the collection of saliva is non-invasive, requires no special equipment, and is suitable for children and elders [27, 28].

The prevalence of the disease in Thailand has never been fully investigated. This study’s objectives are to determine the prevalence of *Leishmania* infection in HIV-infected Thai patients from southern Thailand through PCR analysis of saliva and blood samples.

## Methods

### Study design

The study was conducted in southern Thailand from June to September 2013. A total of 316 HIV-infected patients who came for HIV treatment were enrolled in the study at the Division of Infectious Diseases of Faculty of Medicine, Prince of Songkla University. One ml of saliva and 3 ml of EDTA blood were collected for PCR detection of *Leishmania* DNA.

### Ethics approval

Informed consent was obtained from all subjects according to protocols approved by the Institutional Review Board on Human Research of the Faculty of Medicine, Chulalongkorn University (COA No. 768/2012).

### Study population

Blood and saliva samples were collected from HIV-infected patients who resided in southern Thailand. A total of 316 HIV-infected patients involved in treatment at the Division of Infectious Diseases were enrolled in the study.

### DNA extraction

One ml of whole saliva was used to extract DNA from the tissue using the Invisorb® Spin Tissue Mini Kit (STRATEC Molecular GmbH, Germany) according to the manufacturer’s instructions. To extract the DNA, 200 μl of EDTA blood and 50 μl of buffy coat were used with the extraction kit, Invisorb® Spin blood Mini Kit (STRATEC Molecular GmbH, Germany). Extracted DNA was eluted in 50 μl of elution buffer. The quantity and quality of the extracted DNA were determined using a Nanodrop 2000c (Thermo Scientific, Singapore). Extracted DNA samples were kept at −80 °C for long-term storage.

### PCR amplification

Amplification was performed in a PCR Mastercycler® pro (Eppendorf, Germany) with conditions as follows; denaturation at 94 °C for 4 min, followed by 40 cycles of 94 °C for 1 min’; 65 °C for 1 min; and 72 °C for 1 min, with the final extension at 72 °C for 7 min. The forward and reverse ITS1 regions of the rRNA of *Leishmania* parasite primers were LeF: 5′ TCC GCC CGA AAG TTC ACC GAT A 3’ and LeR: 5′ CCA AGT CAT CCA TCG CGA CAC G 3’, respectively [29]. In order to maintain that the template DNA had been extracted properly, primers that anneal to human DNA (UNFOR403: 5’-TGA GGA CAA ATA TCA TTC TGA GG-3’ and UNREV1025: 5’-GGT TGT CCT CCA ATT CAT GTT A-3’) were used [30]. Therefore, clinical samples which contain human DNA should show the PCR products of 628 bps. The products were analyzed on 1.5 % agarose gel electrophoresis, stained with 0.5 μg/ml ethidium bromide and visualized with Quantity One quantification analysis software, version 4.5.2 Gel Doc EQ system (Bio-Rad, USA). DNA from cultured *Leishmania* promastigotes isolated from a patient [5] was used as the positive control. DNA from saliva and EDTA blood from a healthy individual who had never traveled into endemic areas were used as negative controls.

### Cloning, sequencing and nucleotide analysis

The study was designed to use cloning for sequencing rather than direct sequencing because the ITS1 primers used in this study can amplify closely *L. martiniquensis* and *L. siamensis* at 379 and 371 bps, respectively. Moreover, PCR products obtained from some reactions contained small amount of DNA, while direct sequencing requires at least 30–50 ng/μl of DNA. Amplified PCR products were ligated into pGEM-T Easy Vector (Promega, USA). The ligated vectors were transformed into DH5α competent cells and screened through the blue-white colony selection system. The suspected positive colonies were cultured for further plasmid DNA extraction using the Invisorb® Spin Plasmid Mini kit (STRATEC Molecular GmbH, Germany), following the manufacturer’s instructions. Purification was performed according to the 1^st^ BASE DNA sequencing system (1^st^ base laboratories, Malaysia) using universal forward T7 primer. Nucleotide sequences were analyzed using the BioEdit Sequence Alignment Editor Version 7.0.9.0. The consensus sequences were compared with available sequence data in GenBank using BLAST search (available at http://blast.ncbi.nlm.nih.gov/Blast.cgi). Sequences obtained from this study were submitted to GenBank to be assigned accession numbers.

### Phylogenetic tree construction

A phylogenetic tree was constructed by Maximum-likelihood method using the Kimura’s 2-parameter model implemented in MEGA6 version 6.06 and the tree was tested using 1000 bootstrap replicates. ITS1 sequences of confirmed *L. martiniquensis* (KM677931) [10] and *L. siamensis* (JX195640) [9] were used to compared with ITS1 sequences of our study. *Bodo caudatus* accession no. AY028450 was used as an outgroup.

### Culture of *Leishmania* parasite

Positive PCR samples were cultured on Schneider’s insect medium (Sigma-Aldrich, USA), which contained 10 % fetal bovine serum, 100 U/ml of penicillin, and 100 μg/ml of streptomycin (Sigma-Aldrich, USA). The samples were then incubated at 25 ± 2 °C. The promastigotes were observed daily under an inverted microscopy (Olympus, Japan).

### Tissue biopsy and staining

A tissue biopsy was performed on an ulcer or nodule from the PCR-positive study patients. Tissue sections were stained with Hematoxylin and Eosin (H&E) and examined under a light microscope (Olympus, Japan) at 100X magnification.

## Results

**Fig. 1:**
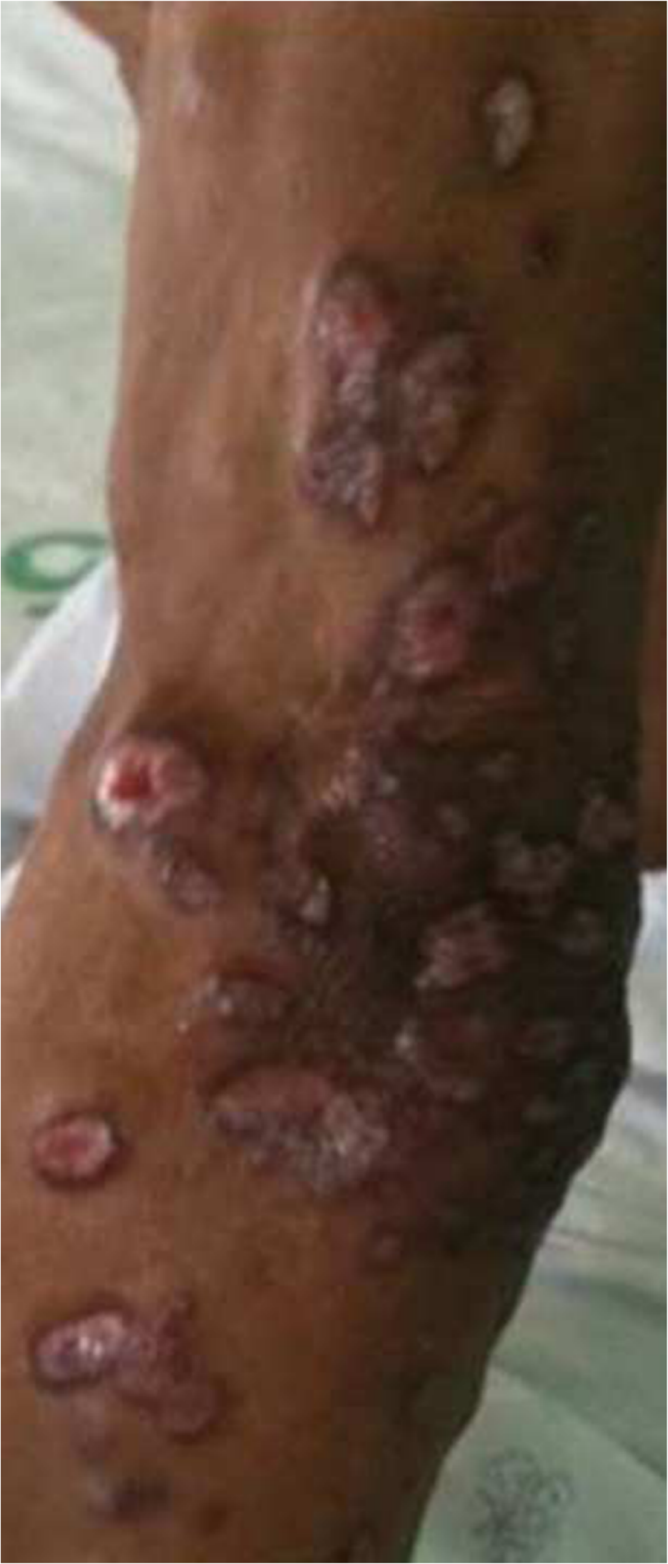
Cutaneous leishmaniasis lesions of the patient 1

Saliva and blood samples were tested with *Leishmania*-specific primers, ITS1 gene by PCR. Three of the 316 saliva samples were positive for *Leishmania* species DNA (0.95 %). Among these three positive cases, two had been diagnosed as leishmaniasis two years previously (Table 1). The first of these cases, involving a 32-year-old male, was diagnosed for disseminated CL 2 years previously. He was treated with amphotericin B deoxycholate and itraconazole, following which his lesions regressed and all samples tested by PCR were negative for *Leishmania* [1–6]. However, in June 2013, he developed multiple papules and ulcers (Fig. 1). A CD4+ T-cell count revealed 110 cells/mm^3^ and he was started on tenofovir, lamivudine and nevirapine for treatment of HIV. Saliva, whole blood, buffy coats, and tissue biopsy were also positive for *Leishmania* DNA. Both culture and H&E stains confirmed the recurrent diagnosis by showing *Leishmania* in a skin biopsy (Table 1).

**Table 1:** Clinical presentations, CD4+ T cell levels, PCR, and Culture/Tissue biopsy for *Leishmania* parasite^a^

Patient	Clinical Presentation	CD4+ T cell count (cells/mm^3^)	Results of PCR for *Leishmania*									Culture/Tissue biopsy for *Leishmania*
			First sample collection			Second sample collection			Third sample collection			
			S	B	BF	S	B	BF	S	B	BF	
32 year old	Relapse disseminated CL 2 years after treatment	110	+	+	+	Not collected			Not collected			+/+
Male												
48 year old	Nodular CL, relapse 2 years after treatment for disseminated CL	207	+	-	-	+	-	-	+	-	+	+/+
Male												
28 year old	Asymptomatic	617	+	-	-	+	-	-	+	-	-	-/N/A
Female												

^a^ *S* Saliva; *B* Blood; *BF* Buffy coat; +: positive; −: Negative; N/A: not available; *CL*: Cutaneous leishmaniasis

**Fig. 2:**
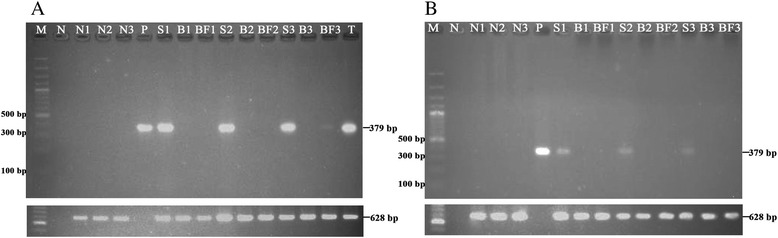
PCR amplification of the ITS1 gene against saliva, buffy coat, blood, and tissue samples of nodular leishmaniasis case (**a**) and asymptomatic case (**b**). PCR amplicons were analyzed by electrophoresis on a 1.5 % agarose gel and stained with ethidium bromide. Lane S1, B1 and BF1: first saliva, blood and buffy coat collection, respectively; lane S2, B2 and BF2:second saliva, blood and buffy coat collection, respectively; and lane S3,B3 and BF3: third saliva, blood and buffy coat collection, respectively; T: tissue, lane M: molecular mass marker (100 basepairs [bp]); lane P: positive control containing extracted DNA from cultured *L. martiniquensis*-produced fragments of 379 bp, lane N: negative control (no DNA template: double-distilled water); lanes N1–N3: negative control (DNA template from non-infected saliva, blood, and buffy coat, respectively); and a PCR for template DNA control shown below (628 bp)

Two other cases were positive only in saliva from the first PCR sample collected (Table 1). One of these cases was a 48-year-old male who was diagnosed two years previously with disseminated leishmaniasis [5] and was treated with liposomal amphotericin B, followed by itraconazole. His clinical status improved and blood and saliva samples were negative after treatment. He received boosted lopinavir and lamivudine for HIV. Blood and saliva samples were collected for a *Leishmania* PCR in July 2013 with only saliva testing positive (Table 1 and Fig. 2a). He developed nodules on his brow, left second toe, left ring finger, and left elbow. His lesions were described by Phumee et al. (2014) [9]. Blood and saliva samples were then collected for two consecutive months (August and September 2013). PCR was positive in buffy coat and saliva samples two months after the first collection (September 2013), (Table 1 and Fig. 2a). A tissue biopsy was performed at a nodule from his brow in September 2013. A PCR of the biopsy sample was positive for the novel *Leishmania* species*.* (Table 1 and Fig. 2a). The first and second cases of leishmaniasis relapsed approximately 2 years after the treatment [5].

The last case was a 28-year-old female who was asymptomatic but whose PCR was positive in saliva (July 2013) and had a CD4+ T-cell count of 617 cells/mm^3^. She did not receive any treatment for HIV. Blood and saliva collected for two consecutive months (August and September 2013) were negative, but PCR remained positive (Table 1 and Fig. 2b).

Amplified sequences obtained from saliva, blood, buffy coat, and tissue of the patient 1 were assigned for accession numbers KU050856-KU050859 respectively. Amplified sequences obtained from saliva, buffy coat, and tissue of patient 2 were assigned for accession numbers KU050860-KU050862, while the amplified sequence from saliva of patient 3 was assigned accession number of KU050863.

**Fig. 3:**
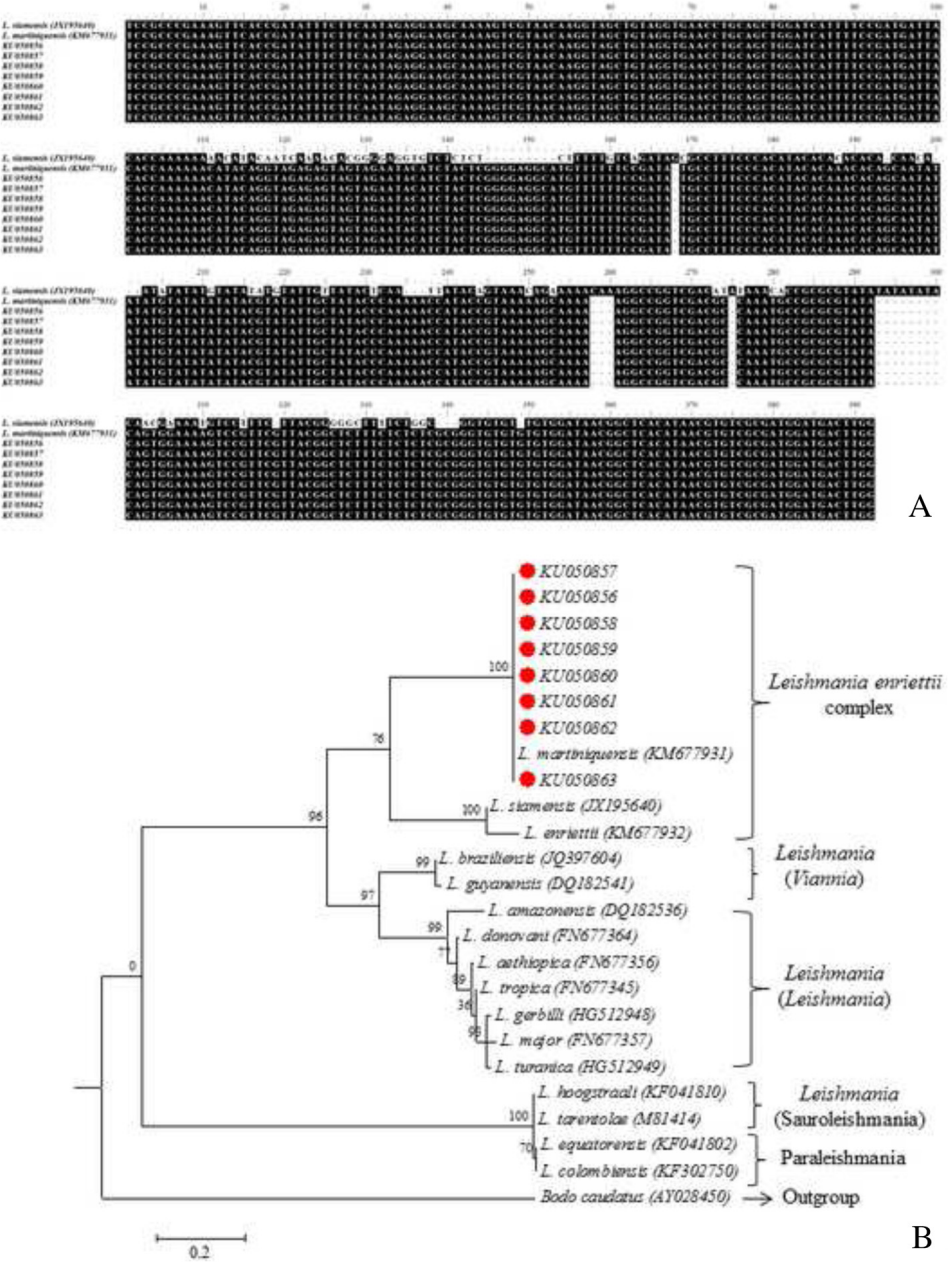
Sequence comparison between *L. martiniquensis* and *L. siamensis*, the different in size and sequences were observed. Red circle indicated *L. martiniquensis* obtained from this study (**a**). A phylogenetic tree showed that both *L. martiniquensis* and *L. siamensis* were classified into *L. enriettii* complex and were discriminate to other *Leishmania* species (**b**)

The nucleotide sequencing of all PCR-positive samples were 100 % identical to *L. martiniquensis* (Fig. 3a and b). The UNFOR403 and UNREV1025 primers which were annealed specifically to human DNA gave positive results for all clinical samples (Fig. 2a and b). This showed that all extracted DNA from clinical samples were extracted properly.

## Discussion

This study identified *Leishmania* co-infections in HIV patients using saliva and blood samples for PCR within an endemic area of Thailand. Three out of 316 saliva samples were positive for PCR Prevalence of *Leishmania* infection in HIV infected patients of this study was 0.95 %. In 2012, Orsini and others investigated prevalence of *Leishmania* infection among 381 HIV-infected patients who living in endemic areas of Brazil. The results showed positive for *Leishmania* in blood samples by using PCR targeted to kDNA region, ELISA, Indirect fluorescent antibody test (IFAT), and rK39 at 6.3 %, 10.8 %, 3.9 %, 0.8 % [30] respectively. Comparing the PCR results, the prevalence of *Leishmania* infection in HIV patients of our study is lower than the result reported by Orsini et al. (2012) [31].

Interestingly in this study, *Leishmania* DNA was detected in saliva prior to appearing in buffy coat in patient two and was also detected only in saliva for patient three. A definite diagnosis was confirmed using microscopy and a culture of tissue biopsy from a nodular lesion. Sequence analysis of amplified PCR products were 100 % identical to *L. martiniquensis* (Accession no KM677931).

Sequence analysis demonstrated that the amplified ITS1 gene region in this study was able to discriminate between *L. martiniquensis* and *L. siamensis* (Fig. 3a). Phylogenetic tree construction showed that both *L. martiniquensis* and *L. siamensis* were classified into the *L. enriettii* complex (Fig. 3b), a result that is similar to that previously reported by Pothirat et al. (2014) [11]. Again, similar to the result of Pothirat et al. (2014) [11] which mentioned that most cases of leishmaniasis in Thailand are caused by *L. martiniquensis*, all three cases of this study were also infected by *L. martiniquensis.*

Patients infected with leishmaniasis in Thailand often have diffuse cutaneous [7, 9], visceral leishmaniasis [1, 3–8] or overlapping diffuse cutaneous and visceral forms [1, 5–9]. Two leishmaniasis cases of this study were presented with cutaneous lesion, one case had diffuse cutaneous lesion (Fig. 1) while another presented with multiple nodular lesions [9].

This study also demonstrated the first asymptomatic *L. martiniquensis* infection in Thailand. Clinical samples were consecutively collected for two months. *Leishmania* DNA was still detected only in the saliva of the asymptomatic case. A study by Phumee et al. (2013) previously demonstrated that *Leishmania* DNA was detected in saliva and buffy coat in all of their cases [1]. However, in this study we found that it was detected only in saliva two months prior to buffy coat, in a patient presenting with nodular leishmaniasis. More recently, Sriworarat et al. (2015) also demonstrated that *L. martiniquensis* DNA was present in saliva prior to blood sample via the use of loop mediated isothermal amplification (LAMP) technique [32]. In this study, *Leishmania* DNA was also detected only in the saliva of an asymptomatic patient. HIV and leishmaniasis co-infection has been previously reported from Thailand [1, 3–9]. Most of these cases were diagnosed from bone marrow or tissue biopsies, and some cases died soon after without therapy [1, 7].

## Conclusions

Our findings showed that early detection of *Leishmania* DNA was found when conducting a PCR from the saliva of two HIV infected patients. This could result in the closer follow up of asymptomatic infected patients and lead to earlier treatment of symptomatic leishmaniasis which could decrease morbidity and mortality rates. This could aid the development of disease surveillance tools, especially in asymptomatic cases therefore improving the design of control strategies. However, before the technique can be deployed, sensitivity and specificity of the test should be evaluated with the larger number of patients.
